# Aberrant Metabolism as Inductor of Epigenetic Changes in Breast Cancer: Therapeutic Opportunities

**DOI:** 10.3389/fonc.2021.676562

**Published:** 2021-10-08

**Authors:** Jossimar Coronel-Hernández, Eloy Andrés Pérez-Yépez, Izamary Delgado-Waldo, Carlos Contreras-Romero, Nadia Jacobo-Herrera, David Cantú-De León, Carlos Pérez-Plasencia

**Affiliations:** ^1^ Laboratorio de Genómica, Instituto Nacional de Cancerología, Mexico City, Mexico; ^2^ Cátedra-CONACYT, Dirección de Cátedras, Consejo Nacional de Ciencia y Tecnología (CONACYT), Mexico City, Mexico; ^3^ Unidad de Bioquímica, Instituto Nacional de Ciencias Médicas y Nutrición, Salvador Zubirán, Mexico City, Mexico; ^4^ Unidad de Investigación en Cáncer, Instituto Nacional de Cancerología , Mexico City, Mexico; ^5^ Laboratorio de Genómica Funcional, Unidad de Biomedicina, Facultad de Estudios Superiores Iztacala, Universidad Nacional Autónoma de México, Mexico City, Mexico

**Keywords:** breast cancer, metabolism, therapeutic targets, epigenetic modifications, glycolysis

## Abstract

Aberrant metabolism is arising interest in the scientific community not only because of the role it plays in the development and establishment of the tumor mass but also the possibility of drug poisoning of key enzymes overexpressed in tumor cells. Moreover, tumor metabolism provides key molecules to maintain the epigenetic changes that are also an undisputed characteristic of each tumor type. This metabolic change includes the Warburg effect and alterations in key pathways involved in glutaminolysis, pentose phosphate, and unsaturated fatty acid biosynthesis. Modifications in all these pathways have consequences that impact genetics and epigenetics processes such as DNA methylation patterns, histone post-translational modifications, triggering oncogenes activation, and loss in tumor suppressor gene expression to lead the tumor establishment. In this review, we describe the metabolic rearrangement and its association with epigenetic regulation in breast cancer, as well as its implication in biological processes involved in cancer progression. A better understanding of these processes could help to find new targets for the diagnosis, prognosis, and treatment of this human health problem.

## Introduction

The progressive process of carcinogenesis induces molecular changes in the cell that enable it to survive in the environment, allowing it to proliferate and grow in unfavorable conditions. Within these changes is the so-called aberrant metabolism. Under standard conditions, normal cells depend on glycolysis to obtain energy; a pathway that triggers the cleavage of glucose to pyruvate. Pyruvate is processed into acetyl-CoA, which is catabolized through a cycle of tricarboxylic acids (TCA) to obtain energy in the form of ATP during oxidative phosphorylation (OXPHOS) (1). On the other hand, tumor cells reprogram the metabolism to satisfy the concentration of essential nutrients and energy, an action known as the Warburg effect (2). This effect consists of a change in obtaining ATP through the degradation of glucose into lactate in the presence of oxygen through enzyme lactate dehydrogenase (LDHA) (3, 4), which enhances ATP production, allowing biosynthesis of biomass to growth and the production of intermediates that promote proliferation and survival (5).

Recent research showed that the aberrant metabolism in cancer is not only involved in maintaining a high proliferative rate or survival but also have consequences that impact epigenetic mechanisms such as DNA methylation, histone post-translational modifications, chromatin remodeler functions, trigger oncogenes activation, and loss in tumor suppressor genes expression to lead the tumor establishment (6). The purpose of this review is to highlight the regulatory implication of the aberrant metabolism in breast cancer over the epigenetic landscape.

## Metabolic Pathways: The Master Regulators of the Gene Expression in Breast Cancer

### Glycolysis and TCA

Glucose is the most abundant catabolite in blood and is the principal primary energy source of cancer cells. Normal cells take glucose from blood vessels and enter the cell through glucose transporter proteins (GLUT). Once in the cytoplasm, it is metabolized into two pyruvate molecules by the glycolysis pathway (7). In breast cancer, the glucose uptake is increased due to the overexpression and translocation of GLUT1 (8) to the cell membrane, enhancing glucose delivery into the cell. This glucose is employed directly for lactate production due to overactivation of AMPK (AMP-activated protein kinase) (9) and overexpression of 6-phosphofructose 2-kinase (6PF2K) (10). In breast cancer cells, pyruvate is usually processed into lactate, which is actively transported to the extracellular matrix due to the upregulation of monocarboxylate 1 (MCT1), an essential lactate transporter, giving them a glycolytic profile. Lactate plays a principal role in regulating gene transcription by inhibiting the HDAC (histone deacetylase) enzymes, promoting hyperacetylation in nucleosomes and active transcriptional state (11). Some reports mention that the histone H4 acetylation levels increase when cells are treated with lactate, promoting changes in gene expression that favors the cancer establishment (12). In breast cancer-associated fibroblasts, the overproduction of lactate induces tumor growth by demethylation of HIF-1α in patients’ tissue (13).

As a result of glycolysis and the metabolic shift orchestrated by the Warburg effect, the pyruvate that is not oxidized into lactate is dehydrogenated by pyruvate dehydrogenase complex (PDC), and it is turned into acetyl-CoA, which enters directly into the tricarboxylic acid cycle (TCA). This cycle, composed of a series of biochemical reactions, has a central role in energy production, macromolecule synthesis, and redox balance (14). In several types of cancer including breast, enzymes that participate in TCA such as isocitrate dehydrogenase (IDH), succinate dehydrogenase (SDH), and fumarate hydratase (FH) are deregulated, affecting enzymes involved in epigenetic processes (15).

Acetyl-CoA is the sole donor of the acetyl group for the acetylation mechanism not only of histones but in general in protein acetylation. It is a central molecule in metabolism as it participates in catabolic (glycolysis and beta-oxidation) and anabolic reactions (lipogenesis, steroid synthesis, acetylcholine synthesis, etc.) (16). HATs (Histone acetyltransferase) transfer the acetyl group from acetyl-CoA to the amino group of lysine in their target proteins to open the chromatin structure. On the other hand, HDACs (Histone desacetyltransferase) catalyze the opposite reaction; the HDACs remove the acetyl group by hydrolysis, modulating the transcriptional repression by closing the chromatin structure (17). Acetylation status could be used as a biomarker to differentiate between breast cancer subtypes. It has been observed, a genomic gain of acetylation of H3K4 in early stages of breast cancer cells, specifically, in genes associated with estrogen response and epithelial-mesenchymal transition (18). The mTOR complex, the principal regulator of cell growth in cancer, also stimulates acetyl-CoA synthesis through ATP citrate lyase hyperphosphorylation (19). Besides, overexpression or copy-number gain of acetyl-CoA synthetase 2 (ACS2) enhances acetyl-CoA production, correlating with breast cancer progression. Moreover, the Warburg effect promoted lipid biosynthesis using acetyl-CoA through acetyl-CoA carboxylase (ACACA) in the MCF7 breast cancer cell line (20). In MCF7, LCCP, and MCF75C cell lines, there was reported a nuclear overactivation of two acetyl-CoA-producing enzymes: PDC and ATP citrate lyase (ACYL); and their repression have a crucial impact on histone acetylation status, proliferation, and endocrine therapy resistance (21, 22).

Another key intermediate is the α-ketoglutarate, which is required as co-substrate for JHDMs (Jumonji C domain-containing histone demethylases) and TET (Ten-eleven translocation) proteins, which participate in histone and DNA demethylation. Also, JHDMs proteins, as JMJD5, interact directly with pyruvate kinase muscle isozymes (PKM) to change the metabolic flux; its inhibition decreases glucose metabolism (23). JMJD4 is considered essential for breast cancer progression given its role in chromosome segregation, enhancing mitotic segregation errors, and triggering cell proliferation (24). All these proteins are overactivated in breast cancer due to α-ketoglutarate being overproduced by glycolysis or glutamine pathway.

### Glutamine Metabolism as a Driver of Epigenetic Changes

Glutamine is a key amino acid that relieves the high growth rates in cancer cells. High amounts of glutamine are utilized for survival and proliferation. This amino acid is required not only for biosynthetic pathways (i.e., nucleic acid synthesis) but also for glutaminolysis, which is converted into TCA cycle intermediates and lactate (25). Thought this way, cancer cells get an extra energy source. The expression of enzymes involved in glutamine metabolism varies widely depending on the cancer type and is affected by tissue of origin and oncogenotypes. The glutamine addiction is suggested to be due to alterations in components of the glutaminolysis pathway in cancer cells. For example, the glutamine uptake principally through solute carrier family 1 neutral amino acid transported member 5 (SLC1A5), also known as ASCT2, is hyperactivated in several types of cancer, leading to the progression and malignancy (26, 27). In head and neck squamous cell carcinoma and breast cancer cells, the inhibition of glutamine transport abolishes cell growth and proliferation and increases apoptosis and autophagy (28). When glutamine is in the cell, it is converted into glutamate by the glutaminase enzyme (GLS). It has been demonstrated that GLS levels correlate with tumor growth rates and malignancy as well as poor prognosis (29). GLS is overexpressed in breast cancer, liver cancer, colorectal cancer, brain cancer, cervical cancer, lung cancer, prostate cancer, and melanoma (30–34). The important role of GLS overexpression in tumor progression is reflected in breast cancer which is related to high-grade tumors and high metastasis rates (35). Also, basal-like triple-negative and HER2+ breast tumors express large amounts of GLS, whereas luminal B tumors have elevated levels than luminal B tumors (36), and the deregulation of glutaminolysis by GLS knockdown induces breast tumor growth inhibition (37). Moreover, the decrease of two alternative transcripts of GLS (KGA and GAC) by alkyl benzoquinones and specific siRNA induces autophagy through mTORC1 inhibition (38). Additionally, it has been widely observed the role of glutamine metabolism in other cellular processes such as purine, pyrimidine, and non-essential amino acid synthesis, fatty acid synthesis, and the support of the effect of reactive oxygen species (ROS) to prevent apoptosis under stress-energy conditions during cancer progression (39). Furthermore, glutaminolysis components regulate signaling pathways that promote tumor growth in breast cancer. The mTOR signaling pathway is activated by glutamate dehydrogenase (GLUD) levels (40), and α-ketoglutarate induces mTOR dimerization and activation (25, 37) to hyperactivated cell proliferation. Also, glutamine fasting induces low levels of STAT3 phosphorylation in high invasive cancer cells (41). For this reason, glutaminolysis inhibition by GLS or GLUD knocking down inhibits migration and invasion and the epithelial-mesenchymal transition (EMT) mediated by STAT3 (42). Alterations in components downstream of the glutaminolysis pathway also induces epigenetic changes that could lead to the repression of anti-oncogenes and trigger cancer progression. It has been demonstrated that mutations of the isocitrate dehydrogenase 1 and 2 induces the conversion of α-ketoglutarate (α-KG) to 2-hydroxyglutarate to inhibit DNA demethylases and histone demethylases, leading to DNA methylation and histone 3 methylation in lysine (K) residues 9, 27, and 20 (43, 44). Also, glutaminolysis regulates histone demethylases as Jumonji domain-containing protein 3 (JMJD3) and ubiquitously transcribed tetratricopeptide repeat X chromosome (UTX) that are specific demethylase of lysine 27 of histone 3 (H3K27). Recently, Bai et al. (45) showed that in absence of glutamine, JMJD3 activity decreases, whereas H3K27me3 levels are increased. It was also demonstrated that JMJD3 interacts with promoter regions of XIAP and survivin, in a glutamine-dependent manner promoting apoptosis resistance in idiopathic pulmonary fibrosis fibroblast. Moreover, the chemical inhibition of GLS induces the diminish of the H3K4me3 mark and increases the acetylation of lysine 16 of histone 4 (H4K16ac) to alter the expression of anti-apoptotic as well as metastatic-associated genes in human breast cancer cells (46, 47). Histone acetylation is another epigenetic mechanism that controls gene expression and regulates cancer development and progression. Due to the significant role of glutaminolysis in cancer biology, the use of several pathway components as therapeutic targets has been proposed (48). However, a glutaminolysis-focused therapy is not available for the clinical management of cancer patients. Therefore, studies that allow us a better understanding of the complexity of glutamine metabolism and its molecular effects in cancer are still needed.

### Lipid Biosynthesis, Lipolysis, and Derived Metabolites

There is an astounding amount of information on the role of hyperactive lipogenesis in the maintenance of tumor progression. The role of lipid membranes in sustaining high rates of cell replication in the tumor mass is evident; however, other functions of key enzymes in lipid biosynthesis have been characterized. Fatty acid synthase (FASN) catalyzes palmitate biosynthesis using acetyl-CoA and malonyl-CoA in the presence of NADPH; FASN is overexpressed in treatment-resistant mammary tumors (49, 50), and fatty acid syntheses are increased in brain metastases in mammary tumors (51). Other functions of FASN in addition to lipid synthesis are mainly associated with oncogenic signaling derived from tyrosine receptor kinases; it has been described that FASN can be directly phosphorylated by HER2, leading to the increased enzymatic activity of FASN enhancing tumor cell invasion and migration (52). One of the most important metabolites in lipid biosynthesis is Acetyl-CoA which is synthesized in the mitochondria *via* various reactions such as oxidative decarboxylation of pyruvate, catabolism of different amino acids, or beta-oxidation of fatty acids, among others. However, since lipid biosynthesis occurs in the cytosol, the generation of Acetyl-CoA is derived mainly from citrate synthesized in the mitochondria and transported to the cytosol where ACL (ATP-Citrate lyase) catalyzes its conversion to Acetyl-CoA (53). In turn, the metabolic pathway responsible for the oxidative degradation of fatty acids is b-oxidation, which provides ATP, NADPH, and acetyl-CoA, and is used in the acetylation of proteins.

Tumor characterizes for a deregulated chromatin architecture. In particular, the cancer stem cells (CSCs) have an open chromatin, where the main donor of acetyl groups to histones is the acetyl-CoA. For instance, histone H4 (H4K8ac, H4K12ac, and H4K16ac) acetylation plays a main role for the maintenance of the stem phenotype of TNBC cells cultured under hypoxic conditions (54). Besides, CSCs have a widely demonstrated participation in drug resistance. Hence the understanding of the metabolic pathways of this type of cells will bring light to different aspects of tumors and their treatment response.

### One Carbon Metabolism and Methylation

Cytosine methylation is the epigenetic modification process most studied since the 1970s. Its mechanism consists of the addition of a methyl group at the 5-position of the cytosine ring catalyzed by DNA methyltransferases. The importance of DNA methylation is that at the promoter level (methylation of CpG islands), hypermethylation promotes silencing of gene expression; whereas global methylation (associated with regions without CpG islands) maintains genomic stability (55, 56). The methylation mechanism occurs not only in DNA but also in RNA and proteins. Nonetheless, the epigenetic role is associated with the methylation of proteins involved in chromatin organization, particularly histones H3 and H4 (57). S-adenosylmethionine (SAM) is the methyl group donor in cellular metabolism; in general, methyl group transfer is catalyzed by methyltransferases (in the case of DNA-by-DNA methyltransferases, DNMTs), which oxidize SAM to S-adenosyl-homocysteine. SAM is the product of the metabolism of one carbon that couples two different cycles, the folate cycle, and the methionine cycle (58). In breast cancer, all the isoforms of DNMT1, 3A, and 3B are overexpressed (59), and overexpression of DNMT3A was associated with poor prognosis in sporadic breast cancer (60). Cancer progression involves chromatin reorganization, a highly complex process in which regions near the promoters of tumor suppressor genes are hypermethylated, inhibiting their transcription; for example, the levels of BRCA-1 and MGMT hypermethylation may not have prognostic value in overall survival (61).

Chromatin organization is also regulated by histone methylation, which occurs at lysine and arginine residues in the tails of histones H3 and H4 (62). In humans, this reaction is catalyzed by histone methyl transferases (HMTs) (63). In breast cancer, an increase in SAM leads to the overactivation of HMTs, allowing the progression of the tumor phenotype (64). The methyltransferase Suv39h1 promotes epithelial-mesenchymal transition by adding the H3K9me3 mark on the E-cadherin promoter (65). Also, Suv39h1 interacts with DNMT1 to hypermethylate the estrogen receptor-alpha (ER) promoter, silencing its expression (66). Therefore, the patterns of both DNA and histone methylation, mediated by aberrant metabolism in breast cancer, are highly relevant to tumor progression.

## Therapeutic Targets

The reprogramming of cellular metabolism is of high relevance in the hallmarks of cancer (67). **Table 1** compiles information on different drugs used to block fundamental enzymes of tumor metabolism. Several reports have shown that alterations in glycolysis, glutamine, lipid, and folate metabolism in breast cancer cells could be used as therapeutic targets. Inhibitors of glycolytic enzymes and transporters of glycolytic products such as GLUT1, hexokinase (HK), 6-phosphofructo 2-kinase-fructose-2, 6-biphosphatase E (PFKFB3), PMK2, LDHA, and monocarboxylate transporter 1 (MCT1) have been studied in numerous preclinical studies (7). The non-metabolizable glucose analog 2-deoxy-D-Glucose (2-DG) blocks the first step in glycolysis. It is phosphorylated by hexokinase to produce 2-DG-6P, which cannot be metabolized, reducing proliferation (78). It has demonstrated that 2-DG exhibits a cytotoxic effect in breast cancer cells with mitochondrial respiratory (79). Another report revealed that 2-DG acts as a radiation and drug sensitizer of breast cancer cells (80). Moreover, a current study using a murine model showed that the combination of 2-DG with oncolytic virotherapy (NDV) induced tumor cell death and inhibited tumor growth (78). At this moment, the effects of this inhibitor in normal and tumor cells of patients remain unexplored; thus, exhaustive clinical studies are desirable.

**Table 1:** Drugs used to target tumor metabolism.

Pathway	Inhibitor	Target molecule	Clinical trial	References
Glycolysis	BAY-876	GLUT1	Preclinical	(68)
Glycolysis	Apple polyphenol phoretin (Ph)	GLUT2	Preclinical	(69)
Glycolysis	Gen-27	Hexokinase II	Preclinical	(70)
Glycolysis	Silibinin	GLUT1	*in vitro*	(71)
Glycolysis	Butyrate	PKM2	Preclinical	(72)
TCA	Butyrate	SIRT3	Preclinical	(73)
Glutaminolysis	l-γ-glutamyl-p-nitroanilide (GPNA)	ASCT2/SLC1A5	Preclinical	(27)
Glycolysis	Oxamate	LDH-A, aspartate aminotransferase	Preclinical	(74)
Glycolysis	Galloflavin	LDH-A	Preclinical	(75)
Glycolysis	Lonidamide	Hexokinase II	Phase II	(76)
Lipid synthesis	Orlistat	Fatty acid synthase	Preclinical	(77)

Glutaminolysis is also considered a potential target in cancer. A critical step in the utilization of glutamine is its conversion to glutamate by the mitochondrial enzyme glutaminase (81). Glutamine analogs as 6-diazo-5-oxo-L-norleucine, azaserine, and acivicin bind irreversibly to the active site of glutaminase, showing antitumoral activity (82). The molecule CB-839, a GLS inhibitor, was tested in cell lines derived from breast cancer tumors showing activity only in triple-negative subtype and not in Her2+ cells (83). But the low potency, poor metabolic stability, and low solubility of these drugs limit their potential for clinical development.

However, *in vitro* and *in vivo* studies have demonstrated that enzymes of lipid metabolism are involved in tumor development and progression, supporting the search for inhibitors to lipids metabolism components. For instance, inhibitors to Fatty Acid Synthase (FAS) as C75, orlistat, C93 have shown effects in stopping tumor growth of xenograft models (77, 84, 85). Nevertheless, these inhibitors have limitations such as low cell permeability, poor solubility, lack of selectivity, among others, precluding the use as a systemic drug (86).

**Figure 1:**
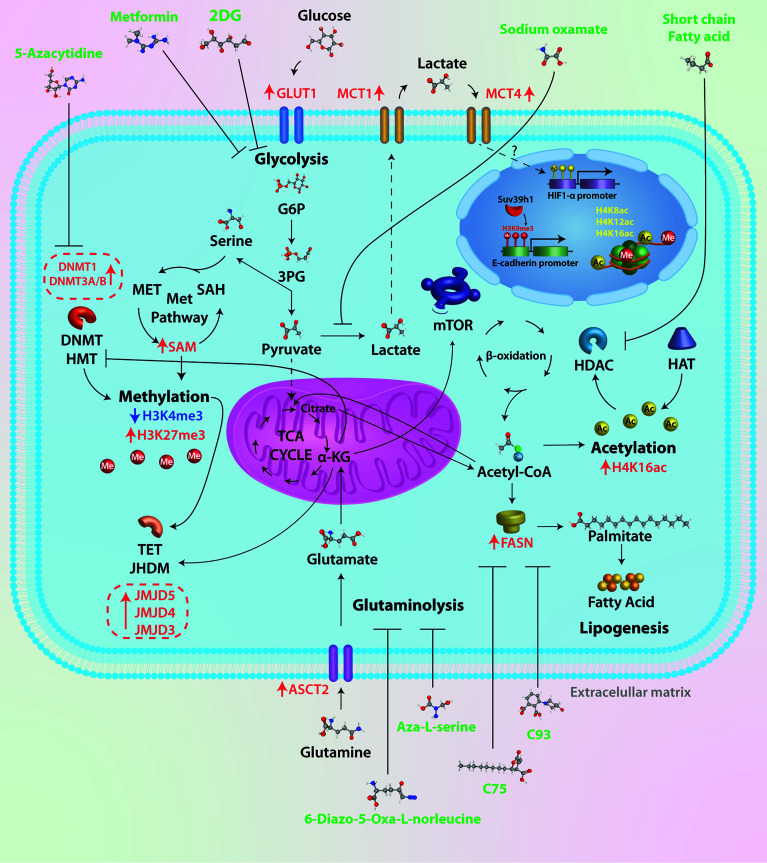
Therapeutic targets of tumor metabolism. Tumor cells have a high glycolytic rate and overexpress key enzymes of glucose metabolism. Several drugs have been used in preclinical trials to test their effectiveness, such as 2DG, a glucose analog whose metabolic product (2-DG-P) is unable to be metabolized, therefore inhibiting glycolysis. Oxamic acid is a competitive inhibitor of LDHA, an enzyme overexpressed in breast cancer cells; the result of this inhibition is lower levels of lactate, a critical oncometabolite for cell migration and tumor progression. In tumor cells, approximately 90% of glucose is metabolized to lactate; to replenish the deficit of carbon molecules, there is an increase in glutamine metabolism. The ASCT2 transporter is overexpressed in breast cancer; glutamine is metabolized in the cytosol to glutamate and subsequently transported to the mitochondrial matrix and incorporated into TCA in the form of α-KG. The antitumor effect of two glutamine analogs (6-diazo-5-oxa-L-norleucine and Aza-L-serine) has been shown. Acetyl-CoA is a central molecule in metabolism as it participates in catabolic (glycolysis and beta-oxidation) and anabolic reactions (lipogenesis, steroid synthesis, acetylcholine synthesis, etc.). In addition to this crucial role in cellular metabolism, Acetyl-CoA is the sole donor of acetyl groups for the acetylation of proteins and particularly histones. Histone acetylation is catalyzed by histone acetyltransferases (HATs), whereas removal of the acetyl group is mediated by histone deacetylases (HDACs). It has been shown that short-chain fatty acids such as valproic acid and butyrate, among others, can inhibit HDACs. Increased levels of acetyl-CoA promote fatty acid synthesis associated with FASN overexpression. Different inhibitors of the key enzyme in fatty acid synthesis have been used (C75, C93, and orlistat, among others), which are inhibitors of the thioesterase domain of fatty acid synthase (FASN). The epigenetic mechanism classically described is DNA methylation. The pathway that supplies methyl groups for both DNA methylation and histone and protein methylation is the one-carbon (1C) pathway metabolism, in which two distinct pathways, the folate and methionine cycle, converge, resulting in the product S-adenosylmethionine. The epigenetic mechanism classically described is DNA methylation. While no inhibitors of these metabolic pathways have been identified, several molecules have been used to inhibit the activity of enzymes involved in DNA methylation (DNMT1, DNMT3/B).

The effect of different drugs with the ability to block tumor metabolism has been studied in breast cancer, such as the combination of metformin and oxamate, mTOR, and LDH-A inhibition, leading to apoptosis and autophagy activation (87). This drug combination dramatically reduced the triple-negative breast tumor growth in mice in a short time, and effectiveness lasted for 5 months after finishing the treatment. These drugs seem to act directly on tumor cells by inhibiting glycolysis and mTOR signaling and activating mechanisms that eventually drive to apoptosis. So, a metabolic shift in breast cancer affects the epigenome directly and has repercussions on gene expression and tumor development. The unraveling of these epigenetic enzymes modulated by the metabolism could serve as pharmacological targets, having a deep impact on the treatment of breast cancer. For a summarized graphical representation of the ideas outlined in this mini-review, see **Figure 1**.

## Author Contributions

Conceptualization: JC-H, EP-Y, CP-P. Writing—Original Draft: CP-P, JC-H, EP-Y, and NJ-H. Preparation: ID-W, CC-R, and DC-L. Writing—Review and Editing: CP-P and NJ-H. All authors contributed to the article and approved the submitted version.

## Conflict of Interest

The authors declare that the research was conducted in the absence of any commercial or financial relationships that could be construed as a potential conflict of interest.

## Publisher’s Note

All claims expressed in this article are solely those of the authors and do not necessarily represent those of their affiliated organizations, or those of the publisher, the editors and the reviewers. Any product that may be evaluated in this article, or claim that may be made by its manufacturer, is not guaranteed or endorsed by the publisher.
